# Effects of hearing intervention on falls in older adults: findings from a secondary analysis of the ACHIEVE randomised controlled trial

**DOI:** 10.1016/S2468-2667(25)00088-X

**Published:** 2025-06

**Authors:** Adele M Goman, Nasya Tan, James Russell Pike, Sarah Y Bessen, Ziheng (Sally) Chen, Alison R Huang, Michelle L Arnold, Sheila Burgard, Theresa H Chisolm, David Couper, Jennifer A Deal, Nancy W Glynn, Theresa Gmelin, Lisa Gravens-Mueller, Kathleen M Hayden, Pablo Martinez-Amezcua, Christine M Mitchell, James S Pankow, Nicholas S Reed, Victoria A Sanchez, Jennifer A Schrack, Kevin J Sullivan, Josef Coresh, Frank R Lin

**Affiliations:** School of Health and Social Care, Edinburgh Napier University, Edinburgh, UK; Cochlear Center for Hearing and Public Health, Johns Hopkins Bloomberg School of Public Health, Baltimore, MD, USA; Cochlear Center for Hearing and Public Health, Johns Hopkins Bloomberg School of Public Health, Baltimore, MD, USA; Department of Population Health and Medicine, NYU Grossman School of Medicine, NYU Langone Health, New York, NY, USA; Department of Epidemiology, Johns Hopkins Bloomberg School of Public Health, Baltimore, MD, USA; Department of Otolaryngology-Head and Neck Surgery, Johns Hopkins University, Baltimore, MD, USA; Cochlear Center for Hearing and Public Health, Johns Hopkins Bloomberg School of Public Health, Baltimore, MD, USA; Cochlear Center for Hearing and Public Health, Johns Hopkins Bloomberg School of Public Health, Baltimore, MD, USA; Department of Otolaryngology-Head and Neck Surgery, Johns Hopkins University, Baltimore, MD, USA; Department of Communication Sciences & Disorders, College of Behavioral and Community Sciences, University of South Florida, Tampa, FL, USA; Gillings School of Global Public Health, University of North Carolina, Chapel Hill, NC, USA; Department of Communication Sciences & Disorders, College of Behavioral and Community Sciences, University of South Florida, Tampa, FL, USA; Gillings School of Global Public Health, University of North Carolina, Chapel Hill, NC, USA; Cochlear Center for Hearing and Public Health, Johns Hopkins Bloomberg School of Public Health, Baltimore, MD, USA; Department of Epidemiology, Johns Hopkins Bloomberg School of Public Health, Baltimore, MD, USA; Department of Otolaryngology-Head and Neck Surgery, Johns Hopkins University, Baltimore, MD, USA; Department of Epidemiology, School of Public Health, University of Pittsburgh, Pittsburgh, PA, USA; Department of Epidemiology, School of Public Health, University of Pittsburgh, Pittsburgh, PA, USA; Gillings School of Global Public Health, University of North Carolina, Chapel Hill, NC, USA; Department of Social Sciences and Health Policy, Wake Forest University School of Medicine, Winston-Salem, NC, USA; Cochlear Center for Hearing and Public Health, Johns Hopkins Bloomberg School of Public Health, Baltimore, MD, USA; Department of Epidemiology, Johns Hopkins Bloomberg School of Public Health, Baltimore, MD, USA; Department of Otolaryngology-Head and Neck Surgery, Johns Hopkins University, Baltimore, MD, USA; Cochlear Center for Hearing and Public Health, Johns Hopkins Bloomberg School of Public Health, Baltimore, MD, USA; Division of Epidemiology and Community Health, University of Minnesota School of Public Health, Minneapolis, MN, USA; Cochlear Center for Hearing and Public Health, Johns Hopkins Bloomberg School of Public Health, Baltimore, MD, USA; Department of Population Health and Medicine, NYU Grossman School of Medicine, NYU Langone Health, New York, NY, USA; Department of Otolaryngology—Head and Neck Surgery, NYU Grossman School of Medicine, NYU Langone Health, New York, NY, USA; Department of Otolaryngology – Head & Neck Surgery, University of South Florida, Tampa, FL, USA; Department of Epidemiology, Johns Hopkins Bloomberg School of Public Health, Baltimore, MD, USA; The MIND Center, University of Mississippi Medical Center, Jackson, MS, USA; Department of Population Health and Medicine, NYU Grossman School of Medicine, NYU Langone Health, New York, NY, USA; Cochlear Center for Hearing and Public Health, Johns Hopkins Bloomberg School of Public Health, Baltimore, MD, USA; Department of Epidemiology, Johns Hopkins Bloomberg School of Public Health, Baltimore, MD, USA; Department of Otolaryngology-Head and Neck Surgery, Johns Hopkins University, Baltimore, MD, USA

## Abstract

**Background:**

Hearing loss is highly prevalent among older adults and has been associated with an increased likelihood of falling. We aimed to examine the effect of a hearing intervention on falls over 3 years among older adults in a secondary analysis of the ACHIEVE study.

**Methods:**

The Aging and Cognitive Health Evaluation in Elders (ACHIEVE) study was a 3-year, unmasked, randomised controlled trial of adults aged 70–84 years at enrolment with untreated hearing loss and without substantial cognitive impairment. Participants were recruited at four US community-based field sites from two study populations: (1) an ongoing observational study of cardiovascular health (Atherosclerosis Risk in Communities [ARIC] study), and (2) de novo from the community. Participants were randomly assigned (1:1) to a hearing intervention (audiological counselling and provision of hearing aids) or a health education control (didactic education and enrichment activities covering chronic disease prevention topics). A prespecified exploratory outcome was falls. Self-reported falls in the past 12 months were assessed at baseline and annually for 3 years, and analysed by intention to treat with covariate adjustment. The study was registered with ClinicalTrials.gov, NCT03243422, and is completed.

**Findings:**

Between Nov 9, 2017, and Oct 25, 2019, 3004 individuals were screened for eligibility and 977 (238 [24%] from the ARIC study and 739 [76%] de novo) were randomly assigned, with 490 (50%) in the hearing intervention group and 487 (50%) in the health education control group. Overall mean age was 76·8 years (SD 4·0), 523 (54%) participants were female and 454 (46%) were male, and 112 (11%) were Black, 858 (88%) were White, and seven (1%) were other race. In adjusted analyses, the intervention group had a 27% reduction in the mean number of falls over 3 years compared with the control group (intervention group: 1·45 [95% CI 1·28 to 1·61]; control group: 1·98 [1·82 to 2·15]; mean difference: −0·54 [95% CI −0·77 to −0·31]). This 3-year effect of hearing intervention was consistent across both the ARIC and de novo study populations.

**Interpretation:**

Hearing intervention versus a health education control was associated with a reduction in the mean number of falls over 3 years in older adults. Ongoing follow-up of ACHIEVE participants in a separate follow-up study (NCT05532657) will enable examination of the longer term effects of hearing intervention on falls.

**Funding:**

US National Institutes of Health.

## Introduction

In the USA, falls were the leading cause of non-fatal injuries in 2023,^1^ and mortality rates from falls among older adults (aged ≥75 years) have more than doubled in the past two decades.^2^ In 2018, more than a quarter of community-dwelling older adults had a fall in the previous year, with one in ten having an injurious fall.^3^ Hearing loss is highly prevalent among older adults and has been associated with an increased likelihood of falling.^4,5^ There are several potential explanations for the association between hearing loss and falling.^6^ First, a common pathological mechanism could lead to concomitant cochlear and vestibular dysfunction as these sense organs are both located in the inner ear. Second, reduced auditory input arising from hearing loss might restrict access to relevant auditory cues needed for auditory and spatial environmental awareness. Third, hearing loss and degraded peripheral auditory encoding could impose a cognitive load for central decoding and reduce the available cognitive resources needed for postural control and navigation of the spatial environment. Finally, hearing loss might also contribute to adverse outcomes such as frailty,^7^ which could potentially mediate the association of hearing loss with increased risk of falls. Importantly, the latter three mechanisms could potentially be modifiable with hearing intervention, but few studies have directly investigated whether hearing intervention can reduce the likelihood of falls among individuals with hearing loss.

Observational cross-sectional and longitudinal studies examining the relationship between hearing aid use and falls have found conflicting results. Some studies have found that hearing aid use is associated with reduced risk of falls,^8–10^ other studies have found that hearing aid use is not associated with a difference in falls compared with no hearing aid use^11^ or limited hearing aid use,^12^ and one longitudinal study found hearing aid use to be associated with an increased risk of falls.^13^ Reasons for these differences between studies could be due to cohort differences, analytical approaches,^14^ the measurement of falls, or the measurement of hearing aid use.

Given that studies to date examining the relationship between hearing aid use and falls are observational, there are concerns of residual confounding when comparing participants who are already hearing aid users to participants who do not use hearing aids. Participants likely differ in both the perceived impact of their hearing loss, as well as in socioeconomic and health care access factors that enable hearing aid ownership and which might also be related to fall risk. To date, no large-scale randomised controlled trial has examined the effect of hearing intervention on falls. The Aging and Cognitive Health Evaluation in Elders (ACHIEVE) study^15^ was a randomised controlled trial of older adults with untreated hearing loss that examined the effect of a hearing intervention versus a health education control on cognitive decline over 3 years. Exploratory data on falls were collected annually. Herein, in a secondary analysis, we investigate the effect of best practice hearing intervention on the rate of total falls, injurious falls, and recurrent falls over 3 years in the ACHIEVE study.

## Methods

### Study design and participants

The ACHIEVE study was an unmasked randomised controlled trial that aimed to investigate the effect of best practice hearing intervention versus a health education control on 3-year cognitive decline in older adults. ACHIEVE is partly nested within the Atherosclerosis Risk in Communities (ARIC) study, an ongoing prospective longitudinal study of older adults. The ARIC study initially enrolled 15 792 adults aged 45–64 years between 1987 and 1989 from a random sample of the surrounding communities at four community-based field sites in the USA (Forsyth County, NC; Jackson, MS; northwest suburbs of Minneapolis, MN; and Washington County, MD). ACHIEVE recruited participants from two populations: (1) adults participating in the ongoing ARIC study (ARIC cohort) and (2) adult volunteers recruited de novo from communities surrounding the ARIC field sites (de novo cohort). De novo participants were recruited through advertisements in local newspaper, radio, and internet advertisements, and related means. Full recruitment procedures have been described previously.^15,16^ Institutional review board approval was obtained from all study sites (Johns Hopkins University [Baltimore, MD], approval number 00008129; University of Mississippi [Jackson, MS], 2017–0227; University of Minnesota [Minneapolis, MN], STUDY00000502; University of North Carolina [Chapel Hill, NC], 17–0971; University of South Florida [Tampa, FL], Pro00032079; and Wake Forest University [Winston-Salem, NC], IRB00043570). The study was registered with ClinicalTrials.gov, NCT03243422, and was completed in 2023. Participants were invited to enrol in a follow-up study (NCT05532657) which is ongoing. The results presented in this Article are from the main trial.

Main inclusion criteria were age 70–84 years with adult-onset bilateral hearing loss, with a better-ear four-frequency (0·5, 1·0, 2·0, and 4·0 kHz) pure-tone average (PTA) threshold of 30 dB or higher and less than 70 dB; word recognition in quiet score at least 60% correct in the better-hearing ear; Mini-Mental State Examination score of at least 23 for those with a high school degree or less, or at least 25 for those with some college education or more; community-dwelling with an intention to remain in the area during the study period; and a fluent English speaker. Main exclusion criteria were self-reported disability in two or more activities of daily living; visual acuity worse than 20/63 on the MNREAD acuity chart (Precision Vision, Woodstock, IL, USA); self-reported hearing aid use in the previous year; medical contraindication to hearing aids; unwillingness to regularly use hearing aids; or permanent conductive hearing loss. The specified audiological criteria identified individuals who would be expected to benefit from amplification and audiological support provided by the hearing intervention. As the primary outcome of the ACHIEVE trial was cognitive decline, the age and Mini-Mental State Examination criteria were specified to allow for recruitment and follow-up of participants who were at risk for cognitive decline but were without substantial cognitive impairment at baseline. All participants provided written informed consent.

### Randomisation and masking

As detailed previously,^15,17^ participants were randomly assigned (1:1) to receive a hearing intervention or a health education control, via permuted block randomisation in varying block sizes^17^ stratified by recruitment source (ARIC or de novo), field site, and severity of hearing loss (better-ear four-frequency PTA <40 dB or ≥40 dB to <70 dB hearing loss]). These PTA thresholds defined mild (<40 dB) and moderate or greater (≥40 dB to <70 dB) hearing loss per WHO criteria at the time of participant enrolment.^18,19^ Eligible spousal or partner pairs were randomly assigned as a unit, stratified by recruitment source and field site. The randomisation allocation schedule was developed by the coordinating centre at the University of North Carolina (Chapel Hill, NC, USA) and completed within the Carolina Data Acquisition and Reporting Tool web-based data management system. Randomisation was unmasked to participants and study staff due to the nature of the hearing intervention (visible hearing aids). To minimise possible bias, the study hypothesis was masked to participants, and participants were informed before randomisation that they would be offered both study interventions (whereby one intervention would be randomly assigned, and the other intervention would be received after 3 years of follow-up). Potential bias was also minimised by use of standardised training protocols for individuals collecting data and assessing outcomes, no access to cognitive testing results from previous study visits for individuals collecting data and the study coordinators, and masking of the study investigators and staff to accumulating trial data (except coordinating centre staff and an unmasked statistician).

### Procedures

The hearing intervention comprised four approximately 1-h one-to-one sessions with an audiologist every 1–3 weeks after randomisation, bilateral hearing aids, the option of additional hearing assistive devices, device use support, and educational materials on self-management and communication strategies.^20^ Follow up one-to-one sessions with an audiologist occurred every 6 months to provide booster support and education. The hearing intervention included the use of real-ear measures to verify the gain and output of the hearing aids.^20^

The health education control intervention matched the hearing intervention in participant contact, with four approximately 1-h one-to-one sessions with a health educator every 1–3 weeks after randomisation, in which educational content on healthy aging from the 10 Keys to Healthy Aging programme were delivered.^21^ The programme is an evidence-based, interactive, health education approach for adults aged 65 years and older on topics relevant to chronic disease and disability prevention. Session content was tailored to each participant. Each session included standardised didactic education (handouts and information about one of the 10 Keys); enrichment activities (setting personal goals and optional extracurricular individual assignments to provide motivation for the participant to engage in the topic); and a 5–10-min upper-body extremity stretching programme. Follow-up one-to-one sessions with a health educator occurred every 6 months to provide booster support and education. After the 3-year follow-up visit, participants in both interventions were offered the other intervention.

Participants completed a baseline assessment before randomisation, which included the full battery of study outcome measures (cognitive assessment, audiometric measures, and functional outcomes)^17^ and were followed up every 6 months for 3 years. Falls were assessed at baseline and at three annual follow-ups visits. Fall occurrence was assessed with the question “In the past 12 months did you fall?”, with binary response options “Yes” or “No”. Participants who responded affirmatively were asked how many times they fell (up to six or more). Fall recurrence was categorised as two or more falls. Participants were asked about the fall they perceived was the most serious with the following questions: “Did you have to limit your activities because you were injured from this fall” and “From this fall, did you have an injury that required you to see your doctor”. An affirmative response to either of these questions was categorised as an injurious fall. Similar fall history questions have been used in other studies.^3^

Recruitment characteristics recorded at baseline were recruitment source, field site, and pair status (recruited with spouse or partner or not). Self-reported demographics included age (years), sex (male or female), race (White, Black, or other), education (less than completed high school, high school graduate or equivalent, or more than high school), income (family income in the past 12 months), and cohabitation status (living alone or not). Demographic information was collected at baseline for the de novo cohort, and from the parent ARIC study for the ARIC cohort.

Health measures recorded at baseline were diabetes (self-reported prescribed medication use for diabetes or self-reported diagnosis by a doctor or other health professional; diabetes type not specified), hypertension (self-reported use of prescribed antihypertensive medication, measured systolic blood pressure ≥140 mm Hg, or measured diastolic blood pressure ≥90 mm Hg), history of stroke (self-reported prescribed medication use for stroke or self-reported diagnosis by a doctor or other health professional), and cigarette smoking status (self-reported as current, former, or never). A global cognition factor score was derived from performance on a neurocognitive test battery assessing executive function, memory, and language using a validated latent variable modelling approach, with higher scores indicating better cognitive function.^22^ Self-reported depressive symptomatology was assessed with the 11-item Center for Epidemiologic Studies Depression Scale (CES-D).^23^ Participants rated each item on a 3-point scale according to how often they felt that way during the past week. Total scores range from 0 to 22 (higher scores indicate greater expression of depressive symptoms). Balance was assessed in a quiet room with the Short Physical Performance Battery (SPPB), a series of physical performance tests designed to assess lower extremity function in older adults.^24,25^ The balance component of the SPPB assesses the ability of maintaining three progressively harder standing positions (side-by-side stand, semi-tandem stand, and tandem stand). The balance score ranges from 0–4, with higher scores representing better performance on the balance tasks. Walking aids were not used for the balance component of the SPPB.

Hearing loss severity at baseline was measured with pure-tone audiometry, defined with the better-ear four frequency PTA threshold and categorised as <40 dB or ≥40 to <70 dB. Self-reported hearing difficulty was assessed with the Hearing Handicap Inventory for the Elderly Screening Version (HHIE-S) which assesses the perceived social and emotional impact of hearing loss.^26^ Individuals rate each of the ten items on a 3-point scale according to if the item affects them. Total scores range from 0–40 (higher scores indicate greater perceived difficulty). Mean daily hours of hearing aid use during the trial were obtained objectively (over the past year from the device data log within the hearing aid manufacturer software), and subjectively (over the past 2 weeks from self-report).

### Outcomes

Falls was a prespecified exploratory outcome, assessed under the remit of physical function in the ACHIEVE study. Falls were assessed in terms of fall occurrence (at least one fall), injurious falls (a fall that resulted in an injury), and fall recurrence (more than one fall) in the past year based on the annual assessments over the 3-year study period.

### Statistical analysis

Given that falls was an exploratory outcome, analyses were considered hypothesis-generating rather than hypothesis-testing. Consequently, we focused on the patterns of effect across outcomes instead of formally evaluating statistical significance.

Baseline participant characteristics were stratified by randomisation group and recruitment source. The total number of falls after randomisation per 1000 person-years was calculated for the intervention and control groups for the intention-to-treat (ITT) population of all randomly assigned participants. The mean number of falls (total, injurious, and non-injurious) per year after randomisation was computed for each participant. Unadjusted and covariate-adjusted linear regression models were used to estimate the ITT effect of randomised treatment assignment on the mean number of falls over 3 years. Wald 95% CIs were generated for adjusted means and adjusted mean differences. When conducting analyses for randomised controlled trials, including baseline covariates that are correlated with the outcome can increase the precision of the average treatment effect.^27^ The covariate-adjusted model included the number of years of follow-up, the number of falls in the year before the baseline assessment, pair status for randomisation, and baseline age, sex, race, recruitment source, field site, education, diabetes, hypertension, history of stroke, smoking status, CES-D score, SPPB balance score, hearing loss severity, HHIE-S score, and global cognition factor score.

In post-hoc ITT analyses, fall occurrence, injury, and recurrence in the past year at the 1-year, 2-year, and 3-year follow-up was estimated using generalised estimating equations (GEE). Time was defined as a categorical variable and an interaction between randomisation and time was specified. Fall occurrence was estimated from a GEE model that used an unstructured covariance matrix and a logit link function. The proportional odds assumption was verified using the Score test. Injurious falls and fall recurrence were estimated from a GEE model that used an independent covariance matrix and a cumulative logit link function. All GEE models adjusted for the number of falls in the year before the baseline assessment, pair status for randomisation, and baseline age, sex, race, recruitment source, field site, education, diabetes, hypertension, history of stroke, smoking status, CES-D score, SPPB balance score, hearing loss severity, HHIE-S score, and the global cognition factor score. An interaction was specified between each covariate and time. Model-based robust variance 95% CIs were generated for all odds ratios (ORs).

Multiple imputation by chained equations was used to generate values for missing baseline measures. Ten imputed datasets were created based on a two-stage analysis^28^ indicating that precision would be maximised by analysing at least four datasets. The imputation model included all variables previously described plus the baseline measures of income and whether the participant lived alone. To mitigate attrition bias, unstabilised and stabilised inverse probability of attrition weights conditional on being alive were calculated using a logistic regression model that incorporated the same variables included in the imputation model. Unstabilised weights were integrated into unadjusted models. Stabilised weights were integrated into covariate-adjusted models. Parameter estimates from models fit to the imputed datasets were combined according to Rubin’s rules.^29^

Sensitivity analyses were done to evaluate the robustness of the results. The propensity of treatment adherence was estimated from a logistic regression model fit to imputed data.^30,31^ Treatment adherence was defined as participants who completed the hearing or health education control intervention (all four intervention sessions), had no major protocol deviations, never wore hearing aids if they were assigned to the control, and never discontinued hearing aid use if they were assigned to the hearing intervention. The propensity model included the same variables used in the imputation model. The estimated propensity of treatment adherence was used to create time-invariant unstabilised inverse probability weights that were integrated into the linear regression models and, post-hoc, into the GEEs, to estimate the complier average causal effect (CACE). A second, post-hoc sensitivity analysis repeated the CACE analysis on intervention effects at each year but used more stringent definitions of treatment adherence that required the participant to use a hearing aid for a minimum mean duration per day of 2, 4, or 6 h. Treatment adherence in the control group in the second sensitivity analysis was unchanged from the definition in the first sensitivity analysis. Analyses for treatment adherence based on hours of daily hearing aid use were conducted for self-reported hearing aid use and for hearing aid use measured by data logging.

All models were performed for the total sample and stratified by recruitment source. Post-hoc stratification by sex was done for the 3-year rate of falls. All analyses were done in SAS (version 9.4).

### Role of the funding source

The funder of the study had no role in study design, data collection, data analysis, data interpretation, or writing of the report.

## Results

Between Nov 9, 2017 and Oct 25, 2019, 3004 individuals were screened for eligibility and 977 (238 [24%] from the ARIC study and 739 [76%] de novo) were randomly assigned, with 490 (50%) in the hearing intervention group and 487 (50%) in the health education control group (appendix p 2, table). Baseline characteristics have been reported in previous publications.^15,16,32^ Briefly, overall mean age was 76·8 years (SD 4·0), 523 (54%) participants were female and 454 (46%) were male, and 112 (11%) were Black, 858 (88%) were White, and seven (1%) were other race. 290 (30%) of 966 participants with available data reported falling in the year before the baseline assessment (table). Within the 3-year study period, there were 625 falls over 1358 person-years in the intervention group (460·2 falls per 1000 person-years), and 837 falls over 1327 person-years in the control (630·7 falls per 1000 person-years) in the ITT population.

Compared with de novo participants, participants recruited from the ARIC study were more likely to be older, female, and Black, have lower education status and lower income, and live alone. Participants from the ARIC study were also more likely to have diabetes or hypertension, and lower global cognition and HHIE-S scores, than de novo participants. In the ITT population, among ARIC participants, there were 361·6 falls per 1000 person-years in the intervention group and 597·7 falls per 1000 person-years in the control group in the ITT population. Among de novo participants, there were 492·2 falls per 1000 person-years in the intervention group and 642·8 falls per 1000 person-years in the control group.

**Figure 1:**
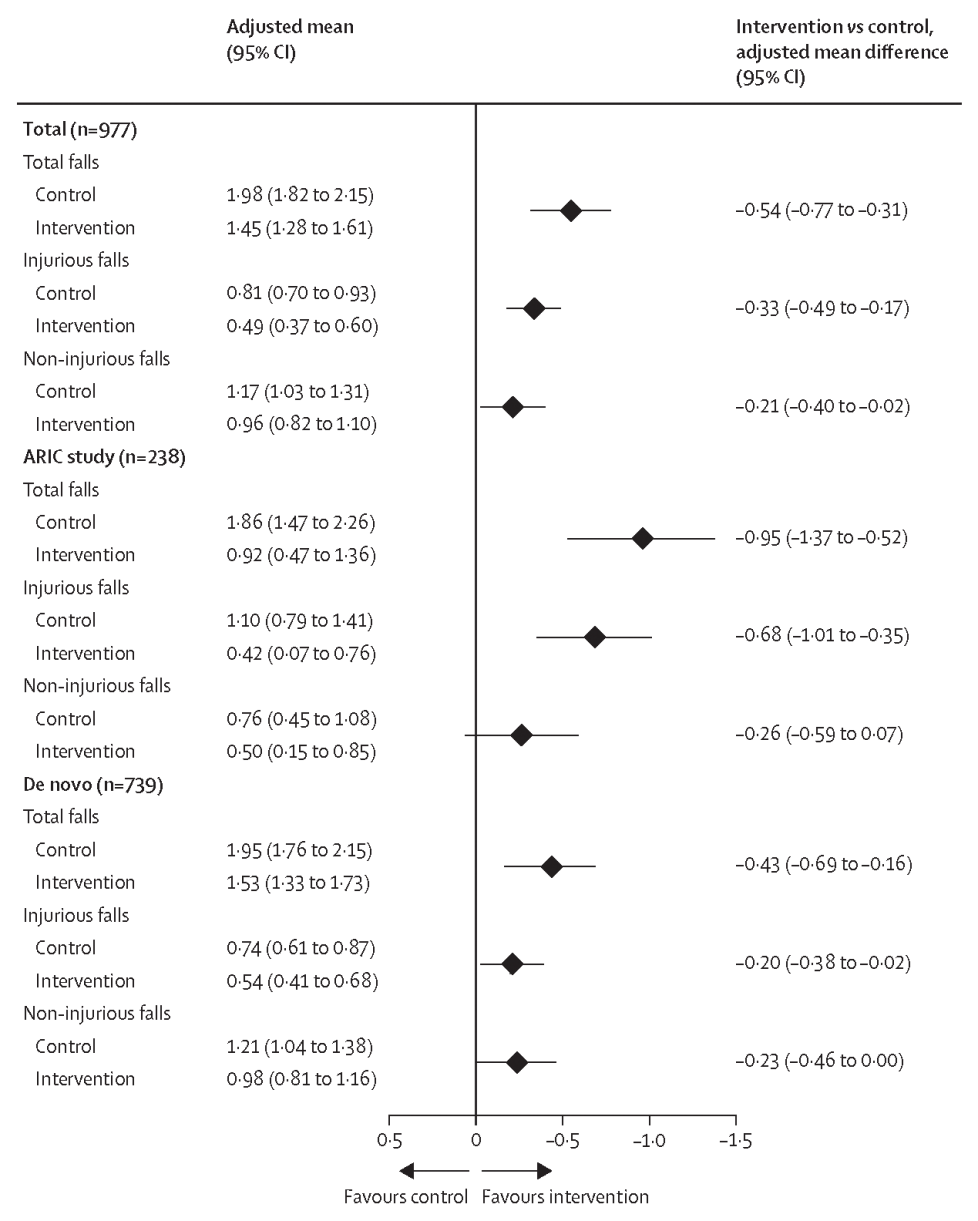
Covariate-adjusted intention-to-treat analysis of the 3-year rate of falls by randomly assigned treatment in the total cohort and stratified by recruitment source Overall means and mean differences were estimated from attrition-weighted linear regression models that examined the mean number of falls over 3 years per participant. The covariate-adjusted model included the number of years of follow-up, the number of falls in the year before the baseline assessment, pair status for randomisation, and baseline age, sex, race, recruitment source, field site, education, diabetes, hypertension, history of stroke, smoking status, 11-item Center for Epidemiologic Studies Depression Scale score, Short Physical Performance Battery balance score, hearing loss severity, Hearing Handicap Inventory for the Elderly Screening Version score, and global cognition factor score. The x-axis shows the adjusted mean difference for the intervention versus control with positive values (favouring the control) to the left and negative values (favouring the intervention) to the right of the vertical line.

In ITT analyses of intervention effect, the mean number of falls over 3 years after adjusting for covariates was 1·45 (95% CI 1·28 to 1·61) in the intervention group and 1·98 (1·82 to 2·15) in the control group (mean difference −0·54 [95% CI −0·77 to −0·31; figure 1), corresponding to a 27% reduction in the mean number of falls over 3 years in the intervention group compared with the control group. The effect was observed in both the ARIC cohort (mean difference −0·95 [−1·37 to −0·52]) and de novo cohort (−0·43 [−0·69 to −0·16]; figure 1), and for both female participants (−0·46 [−0·74 to −0·18]) and male participants (−1·29 [−1·76 to −0·82]; appendix p 7).

A similar effect was observed for injurious falls (figure 1). In ITT analyses, the mean number of injurious falls over 3 years after adjusting for covariates was 0·49 (95% CI 0·37 to 0·60) in the intervention group and 0·81 (0·70 to 0·93) in the control group (mean difference −0·33 [95% CI −0·49 to −0·17]), with an effect observed in both the ARIC cohort (mean difference −0·68 [−1·01 to −0·35]) and de novo cohort (−0·20 [−0·38 to −0·02]).

Results from CACE sensitivity analyses suggested that the average treatment effect (main analysis; figure 1) and the average treatment effect among the treated (CACE analysis; appendix p 4) were similar for the 3-year rate of falls (main estimate of mean difference −0·54 [95% CI −0·77 to −0·31] *vs* CACE estimate −0·42 [−0·71 to −0·14]) and 3-year rate of injurious falls (−0·33 [−0·49 to −0·17] *vs* −0·33 [−0·54 to −0·13]).

**Figure 2:**
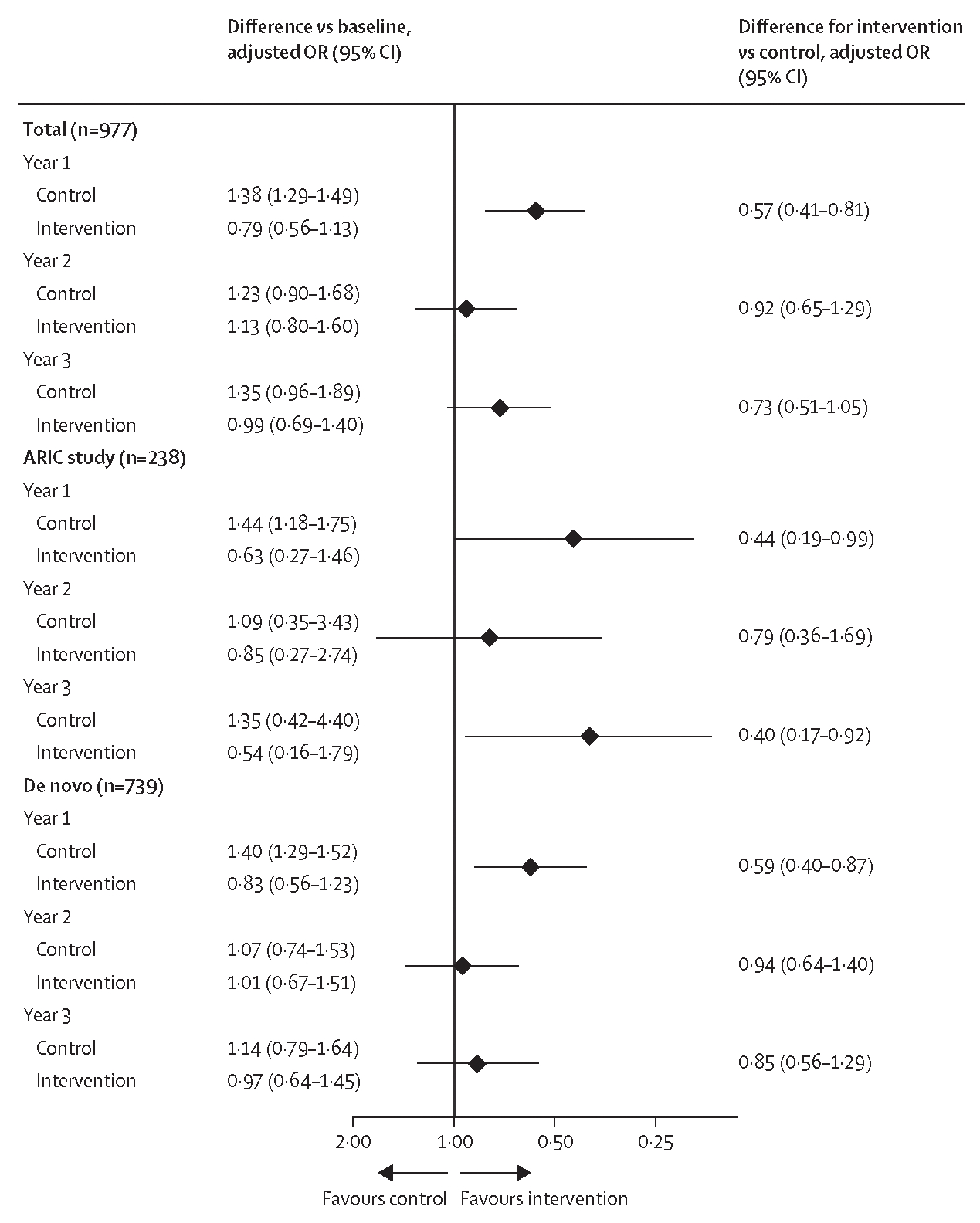
Covariate-adjusted intention-to-treat analysis of change in fall occurrence by randomly assigned treatment in the total cohort and stratified by recruitment source ORs were estimated from attrition-weighted generalised estimating equations of falls occurring after randomisation that were reported at each follow-up year. The model adjusted for the number of falls in the year before the baseline assessment, pair status for randomisation, and baseline age, sex, race, recruitment source, field site, education, diabetes, hypertension, history of stroke, smoking status, 11-item Center for Epidemiologic Studies Depression Scale score, Short Physical Performance Battery balance score, hearing loss severity, Hearing Handicap Inventory for the Elderly Screening Version score, and global cognition factor score. Interactions between randomisation and time, and between time and each covariate, were specified. The x-axis shows the adjusted OR for the intervention versus control with values greater than 1 (favouring the control) to the left and values less than 1 (favouring the intervention) to the right of the vertical line; the x-axis scale is not linear with values above 1 compressed for presentation purposes. OR=odds ratio.

In post-hoc analyses assessing intervention effects at each year of follow-up by ITT, covariate-adjusted GEE models indicated an effect of the hearing intervention versus the health education control on the odds of having a fall in the past year at the 1-year follow-up (OR 0·57 [95% CI 0·41–0·81]; figure 2). The size of the effect was attenuated at the 2-year follow-up (OR 0·92 [0·65–1·29]) and 3-year follow-up (OR 0·73 [0·51–1·05]). The same pattern was observed in the de novo cohort with a beneficial effect observed at the 1-year follow-up (OR 0·59 [0·40–0·87]) that decreased in magnitude at the 2-year follow-up (OR 0·94 [0·64–1·40]) and 3-year follow-up (OR 0·85 [0·56–1·29]). In the ARIC cohort, the odds of falling in the past year were lower in the intervention group versus the control group at the 1-year follow-up (OR 0·44 [0·19–0·99]) and 3-year follow-up (OR 0·40 [0·17–0·92]; figure 2).

**Figure 3:**
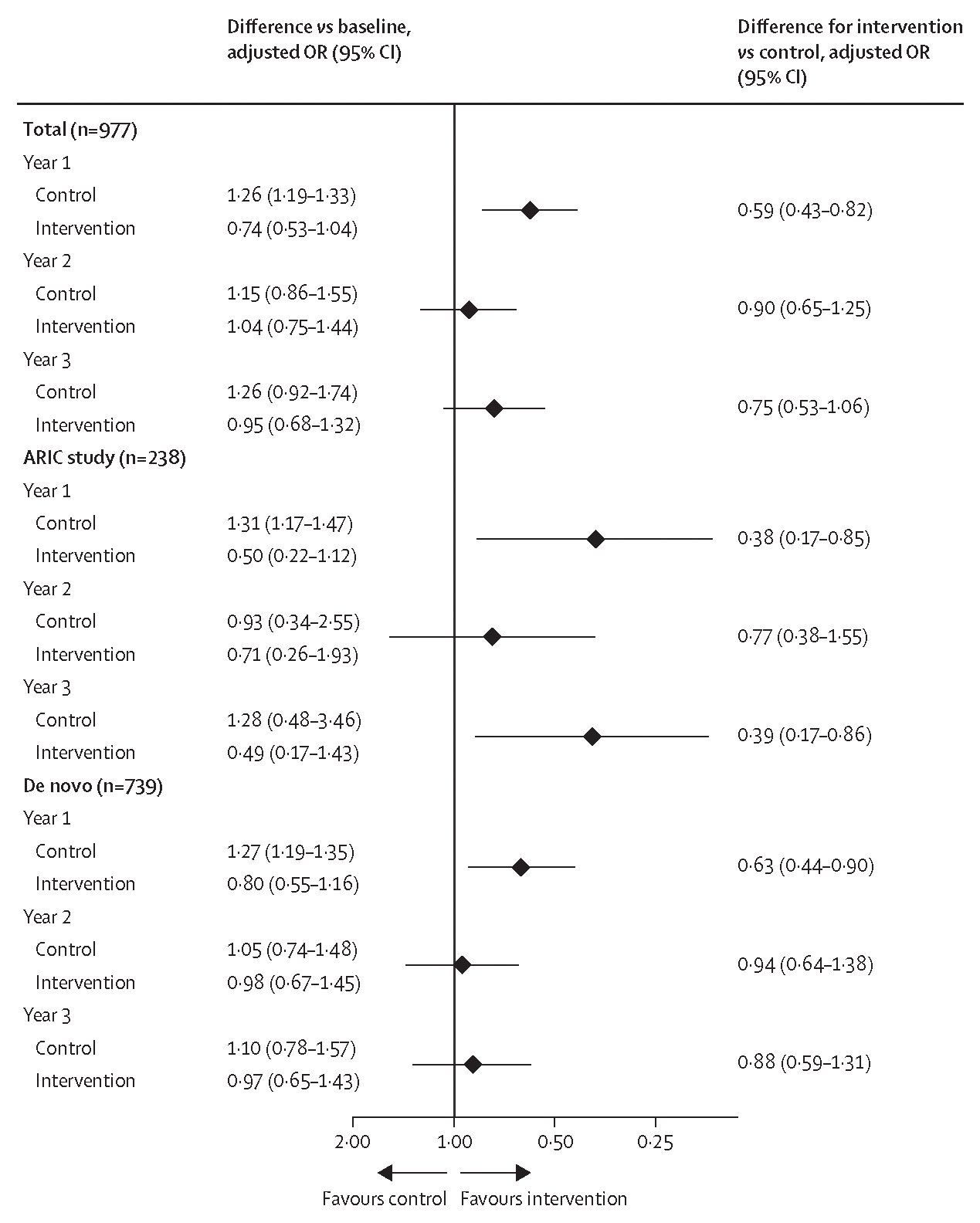
Covariate-adjusted intention-to-treat analysis of change in injurious falls by randomly assigned treatment in the total cohort and stratified by recruitment source ORs were estimated from attrition-weighted generalised estimating equations of falls occurring after randomisation that were reported at each follow-up year. The model adjusted for the number of falls in the year before the baseline assessment, pair status for randomisation, and baseline age, sex, race, recruitment source, field site, education, diabetes, hypertension, history of stroke, smoking status, 11-item Center for Epidemiologic Studies Depression Scale score, Short Physical Performance Battery balance score, hearing loss severity, Hearing Handicap Inventory for the Elderly Screening Version score, and global cognition factor score. Interactions between randomisation and time, and between time and each covariate, were specified. The x-axis shows the adjusted OR for the intervention versus control with values greater than 1 (favouring the control) to the left and values less than 1 (favouring the intervention) to the right of the vertical line; the x-axis scale is not linear with values above 1 compressed for presentation purposes. OR=odds ratio.

Similar patterns were observed in post-hoc covariate-adjusted GEE models that examined injurious falls (figure 3) and recurrent falls (appendix p 3). In the total cohort, the hearing intervention was associated with lower odds of injurious falls versus the health education control at the 1-year follow-up (OR 0·59 [95% CI 0·43–0·82]), but the effect was attenuated at the 2-year follow-up (OR 0·90 [0·65–1·25]) and 3-year follow-up (OR 0·75 [0·53–1·06]). The same pattern was observed in the de novo cohort. In the ARIC cohort, reduced odds of an injurious fall in the intervention group were observed at the 1-year follow-up (OR 0·38 [0·17–0·85]) and 3-year follow-up (OR 0·39 [0·17–0·86]; figure 2). For recurrent falls, in the total cohort, the hearing intervention was associated with lower odds of recurrent falls versus the health education control at the 1-year follow-up (OR 0·59 [95% CI 0·43–0·82]) and 3-year follow-up (OR 0·70 [0·49–0·99]) but not at the 2-year follow-up (OR 0·91 [0·65–1·27]; appendix p 3). The same pattern was observed in the ARIC cohort. In the de novo cohort, the hearing intervention was also associated with lower odds of recurrent falls at the 1-year follow-up (OR 0·62 [0·43–0·89]), but with attenuated effects at the 2-year follow-up (OR 0·95 [0·65–1·39]) and 3-year follow-up (OR 0·82 [0·55–1·24]).

Results from post-hoc CACE sensitivity analyses assessing intervention effects at each year suggested that the average treatment effect (main analysis; figure 2) and the average treatment effect among the treated (CACE analysis; appendix p 5) were similar at the 1-year follow-up (for fall occurrence, main analysis OR 0·57 [95% CI 0·41–0·81] *vs* CACE analysis OR 0·54 [0·37–0·77]), 2-year follow-up (OR 0·92 [0·65–1·29] *vs* 0·94 [0·67–1·32]), and 3-year follow-up (OR 0·73 [0·51–1·05] *vs* 0·72 [0·50–1·02]). Post-hoc sensitivity analyses utilising more stringent definitions of treatment adherence based on hours of daily hearing aid use did not substantially alter this interpretation but the estimates from these models had less precision (appendix p 6).

## Discussion

In this secondary analysis of the ACHIEVE study, we observed that hearing intervention versus a health education control was associated with a reduced rate of falls among older adults. Compared with the control group, participants in the intervention group had a 27% reduction in the mean number of falls over the 3-year study period. This effect was consistent across both the ARIC and de novo study populations that comprised the ACHIEVE cohort, although the effect was qualitatively larger for the ARIC cohort. Speculatively, the difference in effect between the two recruitment sources might be attributable to the ARIC cohort having more risk factors for falls (eg, older age and higher prevalence of hypertension) and therefore might have had a greater intervention benefit.

The existing evidence on the effect of hearing aids on falls is mixed and limited by observational data from non-randomised cross-sectional,^8,33,34^ retrospective cohort,^9,10^ or longitudinal^11,12,13^ studies. A beneficial effect of hearing aid use on fall outcomes has been observed in some observational studies. A cross-sectional US study of 299 adults aged 60 years and older with audiometrically measured hearing loss found that hearing aid users had lower odds versus non-users of having a self-reported fall in the previous 6 months.^8^ In a retrospective cohort study of 114 862 adults aged 66 years and older with hearing loss diagnoses enrolled in a US private health insurance database, it was observed that individuals who had hearing aids (identified by hearing aid procedure codes) had a lower risk of injurious falls over 3 years than individuals with hearing loss who did not have hearing aids.^9^ Another retrospective cohort study examined inpatient admissions among adults older than 18 years at four hospital sites across a 1-year period, and found that patients with self-reported hearing loss without hearing aids were more likely to fall within the hospital setting than patients with hearing loss who did have hearing aids.^10^

Other studies have observed no difference in fall outcomes between individuals with hearing aids and those without hearing aids. A longitudinal study of participants aged 70–79 years found no effect of hearing aid use on the risk of self-reported falls among 407 adults with moderate or greater hearing loss (better-ear four-frequency PTA >40 dB) measured with audiometry.^11^ A 10-year longitudinal study compared self-reported falls between individuals who had at least 2 years of hearing aid use and individuals with no hearing aid use or fewer than 2 years of hearing aid use, and found no difference in the odds of having a fall.^12^ A worsening in fall outcomes has also been observed: an Australian population-based cohort study of 1478 adults aged 55 years and older observed an increased risk of falls among users of hearing aids versus non-users over a 5-year follow-up period.^13^

Methodological differences (including the assessment of falls, the categorisation of hearing aid use, and length of follow-up) and cohort differences (including age, insurance status, and severity of hearing loss) are likely to account for the discrepant findings. The characteristics of the hearing aid users in these previous studies might have differed from non-users, for instance in their perceived hearing difficulty, comorbidities, and resources available to seek health care, which might also be related to falls. ACHIEVE is the first study, to our knowledge, to look at the effect of a hearing intervention on falls in the context of a randomised trial. The apparent beneficial effect of hearing intervention on reducing falls might be due to improved auditory input, enhancing access to relevant auditory cues needed for auditory and spatial environmental awareness and therefore reducing the likelihood of falls. Another possible explanation for the beneficial effect observed is that enhanced auditory input requires fewer cognitive resources for processing, allowing for increased cognitive and attentional resources to be used to maintain postural control and navigate the spatial environment successfully.^6^

Despite a randomised controlled trial design, our study has limitations. Falls were an exploratory outcome of the ACHIEVE randomised controlled trial, and although analyses were prespecified, we consider these results from a secondary analysis of ACHIEVE to be hypothesis-generating rather than hypothesis-testing. Falls were self-reported and therefore subject to incomplete recall. Perceptions of what constitutes a fall can differ across individuals and might have resulted in under-reporting or over-reporting of falls. Although previous research assessing falls among women aged 70 years and older suggested that recalling falls from the previous 12 months has high specificity, it can lack sensitivity.^35^ Additionally, it is possible that COVID-19 pandemic-related restrictions in movement resulted in participants engaging in fewer fall-risk activities, which would have lessened potential differences between the two groups. The year 2 visits were most substantially impacted by pandemic-related restrictions that occurred from March, 2020, through to mid-2021, with no effects of the hearing intervention on falls observed at year 2. It is also possible that participants assigned to the health education control had different falls than they would have had they not participated in the intervention (eg, no contact control), which would also have lessened potential differences between the two groups. Of note, the 10 Keys to Healthy Aging applied as the health education control included a Key on maintaining bones, joints, and muscles, which incorporated aspects related to falls prevention, specifically around identifying fall risks in the home and avoiding behaviours that put one at risk for falls. Participants assigned to the health education control completed this Key at or after the year 1 assessment, with the majority of participants (414 [93%] of 445 who completed the Key) completing this Key at the 18-month appointment and thus before the year 2 falls assessment. As the strongest hearing intervention effect was observed at the 1-year follow-up in the overall study population, the health education control might have had a positive effect on falls in the control group after delivery of this session, and therefore reduced the differences observed between the two groups at 2 and 3 years. Although wider evidence indicates uncertain effectiveness of education for falls prevention, ^36^ it is possible that the effect of hearing intervention on falls might have been different had the control intervention not included aspects explicitly focused on fall prevention.

In conclusion, the current study suggests that a hearing intervention might reduce the average rate of falls over 3 years, which should be confirmed in future studies. The ongoing follow-up of ACHIEVE participants (NCT05532657) will enable the longer term effects of hearing intervention on health outcomes including falls to be examined.

## Supplementary Material

1

## Figures and Tables

**Table:** Characteristics of ACHIEVE participants stratified by randomisation group and recruitment source (intention-to-treat population)

	Total			ARIC study			De novo		
	All (n=977)	Intervention (n=490)	Control (n=487)	All (n=238)	Intervention (n=120)	Control (n=118)	All (n=739)	Intervention (n=370)	Control (n=369)

**Baseline demographic and health characteristics**									
Age, years (n=977)	76·8 (4·0)	76·8 (3·9)	77·0 (4·0)	78·9 (2·9)	79·2 (2·9)	78·6 (2·9)	76·1 (4·0)	75·7 (3·8)	76·5 (4·2)
Sex (n=977)									
Female	523 (54%)	264 (54%)	259 (53%)	147 (62%)	74 (62%)	73 (62%)	376 (51%)	190 (51%)	186 (50%)
Male	454 (46%)	226 (46%)	228 (47%)	91 (38%)	46 (38%)	45 (38%)	363 (49%)	180 (49%)	183 (50%)
Race (n=977)									
Black	112 (11%)	53 (11%)	59 (12%)	68 (29%)	33 (28%)	35 (30%)	44 (6%)	20 (5%)	24 (7%)
White	858 (88%)	434 (89%)	424 (87%)	169 (71%)	86 (72%)	83 (70%)	689 (93%)	348 (94%)	341 (92%)
Other	7 (1%)	3 (1%)	4 (1%)	1 (<1%)	1 (1%)	0	6 (1%)	2 (1%)	4 (1%)
Field site (n=977)									
Forsyth County, NC	236 (24%)	117 (24%)	119 (24%)	61 (26%)	31 (26%)	30 (25%)	175 (24%)	86 (23%)	89 (24%)
Jackson, MI	243 (25%)	120 (24%)	123 (25%)	63 (26%)	30 (25%)	33 (28%)	180 (24%)	90 (24%)	90 (24%)
Minneapolis suburbs, MN	236 (24%)	120 (24%)	116 (24%)	43 (18%)	21 (18%)	22 (19%)	193 (26%)	99 (27%)	94 (25%)
Washington County, MD	262 (27%)	133 (27%)	129 (26%)	71 (30%)	38 (32%)	33 (28%)	191 (26%)	95 (26%)	96 (26%)
Education (n=976)									
Less than completed high school	37 (4%)	19 (4%)	18 (4%)	22 (9%)	12 (10%)	10 (8%)	15 (2%)	7 (2%)	8 (2%)
Completed high school, GED, or vocational school	418 (43%)	206 (42%)	212 (44%)	96 (41%)	48 (40%)	48 (41%)	322 (44%)	158 (43%)	164 (44%)
Some college, graduate, or professional school	521 (53%)	264 (54%)	257 (53%)	119 (50%)	59 (50%)	60 (51%)	402 (54%)	205 (55%)	197 (53%)
Income, US$ (n=950)									
<$25000	147 (15%)	73 (15%)	74 (16%)	60 (27%)	29 (25%)	31 (28%)	87 (12%)	44 (12%)	43 (12%)
$25 000–49 999	283 (30%)	156 (33%)	127 (27%)	77 (34%)	47 (41%)	30 (27%)	206 (28%)	109 (30%)	97 (27%)
$50 000–74 999	210 (22%)	91 (19%)	119 (25%)	47 (21%)	22 (19%)	25 (23%)	163 (22%)	69 (19%)	94 (26%)
$75 000–100 000	140 (15%)	68 (14%)	72 (15%)	21 (9%)	8 (7%)	13 (12%)	119 (16%)	60 (16%)	59 (16%)
>$100 000	170 (18%)	90 (19%)	80 (17%)	20 (9%)	8 (7%)	12 (11%)	150 (21%)	82 (23%)	68 (19%)
Living alone (n=968)	290 (30%)	153 (32%)	137 (28%)	83 (36%)	44 (38%)	39 (34%)	207 (28%)	109 (30%)	98 (27%)
Diabetes (n=977)	195 (20%)	104 (21%)	91 (19%)	68 (29%)	36 (30%)	32 (27%)	127 (17%)	68 (18%)	59 (16%)
Hypertension (n=974)	651 (67%)	333 (68%)	318 (66%)	169 (72%)	87 (73%)	82 (71%)	482 (65%)	246 (66%)	236 (64%)
History of stroke (n=973)	79 (8%)	41 (8%)	38 (8%)	23 (10%)	13 (11%)	10 (8%)	56 (8%)	28 (8%)	28 (8%)
Cigarette smoking status (n=977)									
Current	25 (3%)	17 (3%)	8 (2%)	10 (4%)	8 (7%)	2 (2%)	15 (2%)	9 (2%)	6 (2%)
Former	443 (45%)	219 (45%)	224 (46%)	97 (41%)	48 (40%)	49 (42%)	346 (47%)	171 (46%)	175 (47%)
Never	509 (52%)	254 (52%)	255 (52%)	131 (55%)	64 (53%)	67 (57%)	378 (51%)	190 (51%)	188 (51%)
CES-D score (n=977)	2·5 (2·5)	2·5 (2·6)	2·5 (2·4)	2·7 (2·7)	2·7 (2·9)	2·7 (2·6)	2·4 (2·5)	2·4 (2·6)	2·4 (2·4)
SPPB balance score (n=967)	3·6 (0·8)	3·7 (0·8)	3·6 (0·8)	3·5 (1·0)	3·5 (1·0)	3·5 (1·0)	3·7 (0·8)	3·7 (0·7)	3·7 (0·8)
Hearing loss severity: pure-tone average, dB (n=977)									
<40	425 (44%)	209 (43%)	216 (44%)	99 (42%)	50 (42%)	49 (42%)	326 (44%)	159 (43%)	167 (45%)
≥40 to <70	552 (56%)	281 (57%)	271 (56%)	139 (58%)	70 (58%)	69 (58%)	413 (56%)	211 (57%)	202 (55%)
HHIE-S score (n=970)	15·3 (9·8)	15·7 (10·7)	14·9 (9·3)	12·0 (9·5)	12·7 (10·8)	11·4 (8·6)	16·3 (9·6)	16·7 (9·9)	16·0 (9·8)
Global cognition (n=977)	0·000 (0·926)	0·012 (0·949)	−0·011 (0·902)	−0·379 (1·042)	−0·411 (1·024)	−0·346 (1·062)	0·123 (0·851)	0·149 (0·883)	0·896 (0·818)
**Number of participants with falls: baseline and follow-up**									
Any falls in the previous year									
Baseline (n=966)	290 (30%)	142 (29%)	148 (31%)	60 (25%)	31 (26%)	29 (25%)	230 (32%)	111 (30%)	119 (33%)
Year 1 (n=925)	271 (29%)	117 (25%)	154 (34%)	52 (23%)	21 (19%)	31 (27%)	219 (31%)	96 (27%)	123 (36%)
Year 2 (n=899)	279 (31%)	136 (30%)	143 (32%)	58 (27%)	26 (24%)	32 (29%)	221 (32%)	110 (32%)	111 (33%)
Year 3 (n=869)	267 (31%)	118 (27%)	149 (34%)	63 (31%)	25 (25%)	38 (36%)	204 (31%)	93 (28%)	111 (34%)
Two or more falls in the previous year									
Baseline (n=966)	128 (13%)	54 (11%)	74 (15%)	28 (12%)	12 (10%)	16 (14%)	100 (14%)	42 (11%)	58 (16%)
Year 1 (n=925)	113 (12%)	48 (10%)	65 (14%)	23 (10%)	10 (9%)	13 (11%)	90 (13%)	38 (11%)	52 (15%)
Year 2 (n=899)	126 (14%)	58 (13%)	68 (15%)	23 (12%)	8 (7%)	15 (14%)	103 (15%)	50 (14%)	53 (16%)
Year 3 (n=869)	129 (15%)	57 (13%)	72 (17%)	31 (15%)	11 (11%)	20 (19%)	98 (15%)	46 (14%)	52 (16%)
Injurious falls in the previous year									
Baseline (n=965)	107 (11%)	48 (10%)	59 (12%)	18 (8%)	9 (8%)	9 (8%)	89 (12%)	39 (11%)	50 (14%)
Year 1 (n=925)	101 (11%)	42 (9%)	59 (13%)	23 (10%)	7 (6%)	16 (14%)	78 (11%)	35 (10%)	43 (13%)
Year 2 (n=899)	97 (11%)	48 (11%)	49 (11%)	16 (7%)	5 (5%)	11 (10%)	81 (12%)	43 (12%)	38 (11%)
Year 3 (n=869)	99 (11%)	45 (10%)	54 (12%)	26 (13%)	9 (9%)	17 (16%)	73 (11%)	36 (11%)	37 (11%)

Data are n (%) or mean (SD). Adapted from Lin et al.^15^ Denominators for percentages are based on the number of participants with complete data, as indicated after each characteristic or timepoint. Age, sex, race, education, cohabitation status, and smoking status were self-reported. Income was self-reported family income over the past 12 months. Diabetes was defined as present if the participant reported use of prescribed diabetes medication or self-reported a medical practitioner (doctor or other health professional) diagnosis of any type of diabetes. Hypertension was defined as present based on self-reported use of prescribed antihypertensive medication, measured systolic blood pressure greater than or equal to 140 mm Hg, or diastolic blood pressure greater than or equal to 90 mm Hg. History of stroke was based on whether the participant reported use of prescribed medication for stroke or self-reported a medical practitioner diagnosis. Hearing loss severity was defined based on four-frequency (0·5, 1·0, 2·0, and 4·0 kHz) pure-tone average in the better ear. Global cognition factor scores were developed using a validated latent variable modelling approach^22^ with higher scores indicating better cognitive function. GED=general educational development credential. CES-D=Center for Epidemiologic Studies Depression Scale. SPPB=Short Physical Performance Battery. HHIE-S=Hearing Handicap Inventory for the Elderly Screening Version.

## Data Availability

A de-identified dataset and data dictionary have been submitted to the US National Heart, Lung, and Blood Institute (NHLBI) Biologic Specimen and Data Repository Information Coordinating Center and will be made publicly available in mid-2025. At that time, additional details on data access policies will be made available at https://www.achievestudy.org. The study protocol and statistical analysis plan are available at https://clinicaltrials.gov/study/NCT03243422.

## Research in context

### Evidence before this study

Previous observational studies examining the relationship between hearing aid use and falls have reported contradictory findings. We searched PubMed on Jan 10, 2025, using the search terms (“Hearing Aids”[Mesh] OR “Aid, Hearing”[tw] OR “Hearing, Aid”[tw] OR “Aids, Hearing”[tw] OR “Hearing, Aids”[tw] OR “Hearing Aid”[tw]) AND (“falls”[tw] OR “Accidental Falls”[Mesh]), for studies in English that examined the effect of hearing aids on falls. No randomised control trials were identified.

### Added value of this study

The Aging and Cognitive Health Evaluation in Elders (ACHIEVE) study was a randomised controlled trial in older adults with untreated hearing loss that examined the effect of a hearing intervention versus a health education control on cognitive decline over 3 years. Exploratory data on falls were collected annually. Analyses of these data indicated that hearing intervention versus control was associated with a reduction in the mean number of falls over 3 years. To our knowledge, the ACHIEVE trial is the first large-scale randomised control trial that has examined the effect of hearing intervention on falls.

### Implications of all the available evidence

Taken together, our findings suggest that a best practice hearing intervention might reduce falls among older adults with hearing loss. Ongoing follow-up of ACHIEVE participants in a separate follow-up study (NCT05532657) will enable examination of the longer term effects of hearing intervention on falls.
