# Supplementary material for: Effects of hearing intervention on falls in older adults: findings from a secondary analysis of the ACHIEVE randomised controlled trial
Source: Lancet Public Health. Author manuscript; Available in PMC 2025 Jun 27. (PMC12203021; doi:10.1016/S2468-2667(25)00088-X)
Supplement: 1 [file NIHMS2085667-supplement-1.pdf]

THE LANCET
Public Health

Supplementary appendix

This appendix formed part of the original submission and has been peer reviewed. We post it as supplied by the authors.

Supplement to: Goman AM, Tan N, Pike JR, et al. Effects of hearing intervention on falls in older adults: findings from a secondary analysis of the ACHIEVE randomised controlled trial. *Lancet Public Health* 2025; **10:** e492–502.

Effects of hearing intervention on falls in older adults: Findings from a secondary analysis of the ACHIEVE trial (Supplementary material)

**Supplemental tables/figures:**	**Page**
eFigure 1. Flow of participants in the ACHIEVE study	2
eFigure 2. Covariate-adjusted intention to treat analysis of change in fall recurrence by randomly assigned treatment among the total cohort and stratified by recruitment source, ACHIEVE study	3
eTable 1. Covariate-adjusted complier average causal effect analyses of the 3-year rate of falls by randomly assigned treatment among the total cohort and stratified by recruitment source, ACHIEVE study	4
eTable 2. Covariate-adjusted complier average causal effect analyses of fall occurrence, fall recurrence, and injurious falls by recruitment source, ACHIEVE study	5
eTable 3. Covariate-adjusted treatment effect of fall occurrence, fall recurrence, and injurious falls by treatment adherence, ACHIEVE study	6
eTable 4. Covariate adjusted intention to treat analysis of the 3-year rate of falls stratified by self-reported sex, ACHIEVE study	7

eFigure 1. Flow of participants in the ACHIEVE study

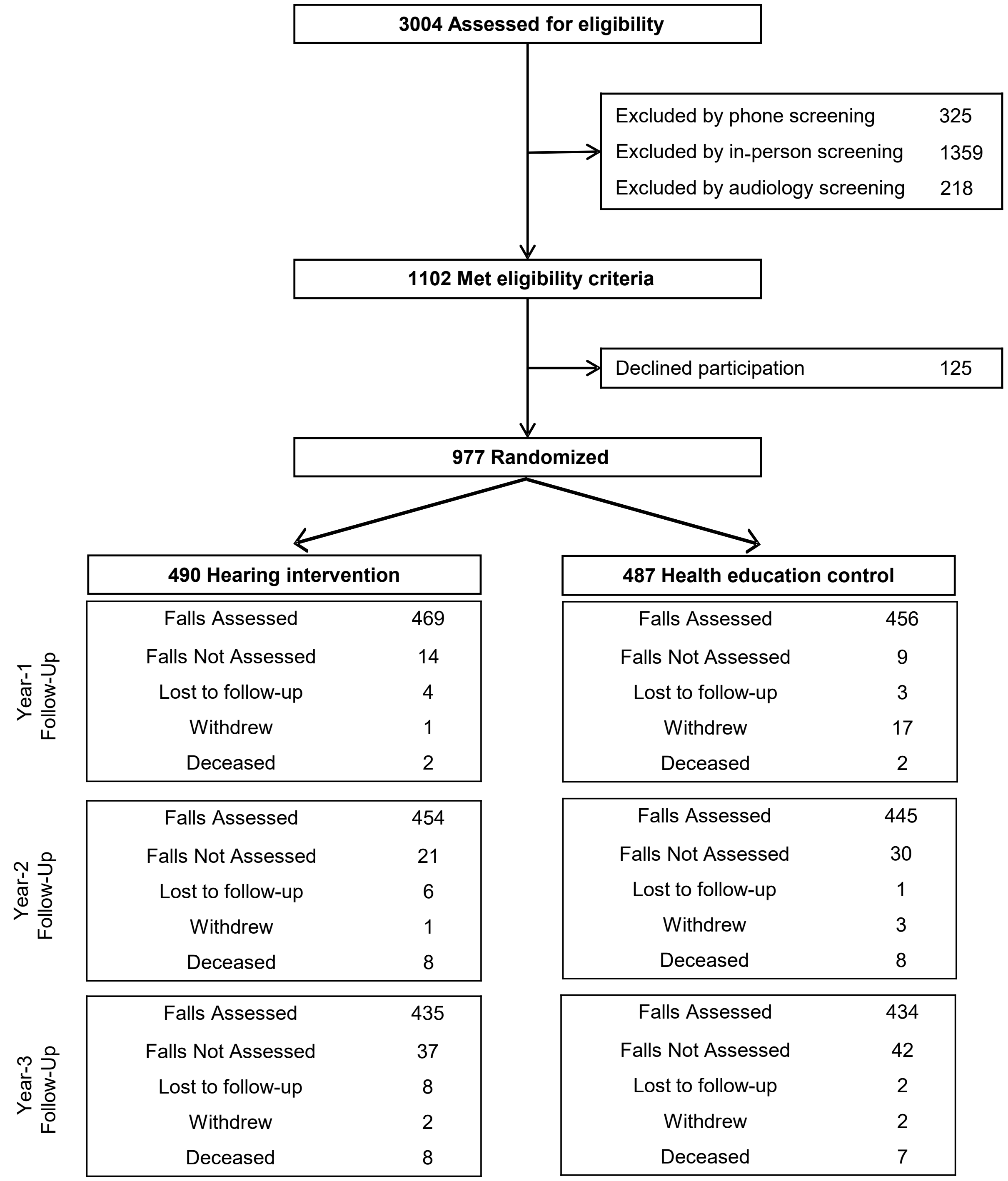

Abbreviations: ACHIEVE = Aging and Cognitive Health Evaluation in Elders. The ACHIEVE study was conducted between 2017-2022 with recruitment from four US community sites.

eFigure 2. Covariate-adjusted intention to treat analysis of change in fall recurrence by randomly assigned treatment among the total cohort and stratified by recruitment source, ACHIEVE study

		Change by Group Adjusted OR (95% CI)	Difference Between Intervention & Control Adjusted OR (95% CI)
**Total (n=977)**			
Year 1	Control	1.42 (1.33, 1.52)	0.59 (0.43, 0.82)
	Intervention	0.84 (0.60, 1.18)	
Year 2	Control	1.34 (0.99, 1.80)	0.91 (0.65, 1.27)
	Intervention	1.22 (0.87, 1.70)	
Year 3	Control	1.51 (1.10, 2.08)	0.70 (0.49, 0.99)
	Intervention	1.06 (0.75, 1.49)	
**ARIC (n=238)**			
Year 1	Control	1.48 (1.26, 1.74)	0.41 (0.19, 0.92)
	Intervention	0.61 (0.27, 1.37)	
Year 2	Control	1.29 (0.45, 3.73)	0.73 (0.36, 1.51)
	Intervention	0.94 (0.32, 2.76)	
Year 3	Control	1.62 (0.54, 4.81)	0.33 (0.15, 0.74)
	Intervention	0.53 (0.17, 1.69)	
**De Novo (n=739)**			
Year 1	Control	1.43 (1.32, 1.55)	0.62 (0.43, 0.89)
	Intervention	0.88 (0.60, 1.29)	
Year 2	Control	1.16 (0.82, 1.63)	0.95 (0.65, 1.39)
	Intervention	1.10 (0.74, 1.64)	
Year 3	Control	1.26 (0.90, 1.78)	0.82 (0.55, 1.24)
	Intervention	1.04 (0.70, 1.55)	

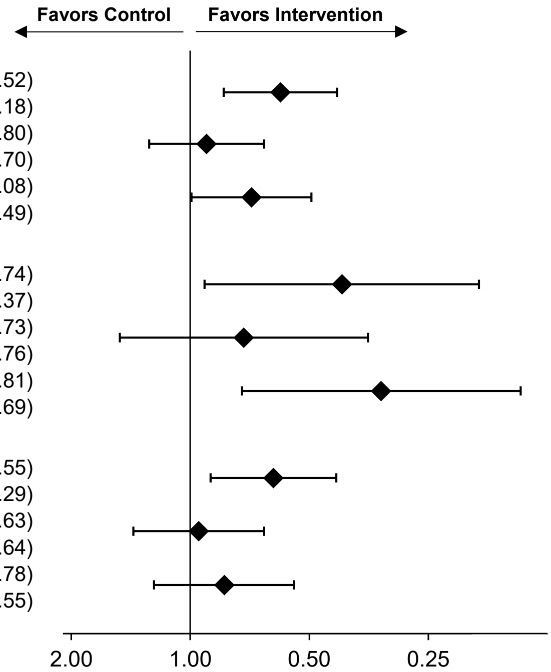

Abbreviations: ACHIEVE = Aging and Cognitive Health Evaluation in Elders; OR = odds ratio; CI = confidence interval.

Estimated from weighted generalized estimating equations of falls occurring after randomization that were reported at each follow-up year (one, two, three). The model adjusted for the number of falls in the year before randomization, pair status, age, sex, race, recruitment source, field site, education, diabetes, hypertension, stroke, smoking status, CES-D score, SPPB balance score, hearing severity, HHIE-S score, and the global cognition factor score. An interaction between time and each covariate was specified. The ACHIEVE study was conducted between 2017-2022 with recruitment from four US community sites.

eTable 1. Covariate-adjusted complier average causal effect analyses of the 3-year rate of falls by randomly assigned treatment among the total cohort and stratified by recruitment source, ACHIEVE study

		**Total**<br>**Mean (95% CI)**	**ARIC**<br>**Mean (95% CI)**	**De Novo**<br>**Mean (95% CI)**
Total Falls	Control	1.94 (1.74, 2.14)	1.89 (1.36, 2.42)	1.83 (1.61, 2.05)
	Intervention	1.51 (1.31, 1.71)	0.91 (0.34, 1.49)	1.59 (1.37, 1.81)
	Difference	-0.42 (-0.71, -0.14)	-0.98 (-1.54, -0.41)	-0.24 (-0.54, 0.07)
Injurious Falls	Control	0.86 (0.71, 1.00)	1.22 (0.83, 1.61)	0.73 (0.57, 0.89)
	Intervention	0.52 (0.38, 0.67)	0.47 (0.04, 0.89)	0.63 (0.47, 0.80)
	Difference	-0.33 (-0.54, -0.13)	-0.75 (-1.18, -0.33)	-0.10 (-0.33, 0.13)
Non-Injurious Falls	Control	1.08 (0.91, 1.25)	0.67 (0.24, 1.11)	1.10 (0.91, 1.29)
	Intervention	0.99 (0.82, 1.16)	0.45 (-0.02, 0.92)	0.96 (0.77, 1.15)
	Difference	-0.09 (-0.33, 0.15)	-0.22 (-0.69, 0.25)	-0.14 (-0.41, 0.13)

Abbreviations: ACHIEVE = Aging and Cognitive Health Evaluation in Elders; CI = confidence interval. Estimated from weighted linear regression models that examined the average number of falls over three-years per participant. The model adjusted for the number of years of follow-up, the number of falls in the year before randomization, pair status, age, sex, race, recruitment source, field site, education, diabetes, hypertension, stroke, smoking status, CES-D score, SPPB balance score, hearing severity, HHIE-S score, and the global cognition factor score. The ACHIEVE study was conducted between 2017-2022 with recruitment from four US community sites.

eTable 2. Covariate-adjusted complier average causal effect analyses of fall occurrence, fall recurrence, and injurious falls by recruitment source, ACHIEVE study

		Fall occurrence			Fall recurrence			Injurious falls		
		Total	ARIC	De Novo	Total	ARIC	De Novo	Total	ARIC	De Novo
		OR (95% CI)	OR (95% CI)	OR (95% CI)	OR (95% CI)	OR (95% CI)	OR (95% CI)	OR (95% CI)	OR (95% CI)	OR (95% CI)
Year 1	Control	1.34 (1.25, 1.44)	1.39 (1.16, 1.68)	1.39 (1.28, 1.50)	1.40 (1.31, 1.49)	1.48 (1.26, 1.74)	1.44 (1.33, 1.55)	1.24 (1.17, 1.31)	1.31 (1.17, 1.47)	1.26 (1.19, 1.34)
	Intervention	0.72 (0.49, 1.05)	0.47 (0.20, 1.12)	0.83 (0.56, 1.23)	0.77 (0.53, 1.11)	0.48 (0.20, 1.13)	0.89 (0.61, 1.30)	0.70 (0.49, 0.99)	0.38 (0.16, 0.90)	0.81 (0.56, 1.18)
	Difference	0.54 (0.37, 0.77)	0.34 (0.14, 0.80)	0.60 (0.41, 0.88)	0.55 (0.39, 0.79)	0.33 (0.14, 0.77)	0.62 (0.43, 0.89)	0.56 (0.40, 0.79)	0.29 (0.12, 0.68)	0.64 (0.45, 0.92)
Year 2	Control	1.19 (0.87, 1.62)	1.16 (0.36, 3.68)	1.05 (0.74, 1.50)	1.32 (0.98, 1.78)	1.44 (0.50, 4.14)	1.15 (0.82, 1.62)	1.15 (0.86, 1.55)	1.03 (0.37, 2.85)	1.05 (0.75, 1.47)
	Intervention	1.11 (0.78, 1.58)	0.90 (0.27, 3.00)	1.05 (0.70, 1.57)	1.23 (0.88, 1.72)	1.06 (0.36, 3.13)	1.16 (0.78, 1.72)	1.05 (0.76, 1.46)	0.79 (0.29, 2.20)	1.03 (0.70, 1.52)
	Difference	0.94 (0.67, 1.32)	0.78 (0.36, 1.68)	0.99 (0.67, 1.46)	0.93 (0.67, 1.30)	0.74 (0.36, 1.50)	1.01 (0.69, 1.47)	0.91 (0.66, 1.27)	0.77 (0.39, 1.54)	0.98 (0.67, 1.43)
Year 3	Control	1.34 (0.96, 1.88)	1.45 (0.44, 4.80)	1.17 (0.81, 1.68)	1.54 (1.12, 2.12)	1.83 (0.61, 5.52)	1.32 (0.94, 1.85)	1.29 (0.94, 1.77)	1.35 (0.50, 3.70)	1.15 (0.81, 1.63)
	Intervention	0.96 (0.67, 1.37)	0.51 (0.15, 1.75)	0.99 (0.66, 1.49)	1.07 (0.76, 1.50)	0.59 (0.19, 1.89)	1.08 (0.73, 1.61)	0.96 (0.69, 1.33)	0.51 (0.18, 1.49)	1.00 (0.68, 1.48)
	Difference	0.72 (0.50, 1.02)	0.35 (0.15, 0.81)	0.85 (0.56, 1.28)	0.69 (0.49, 0.98)	0.32 (0.15, 0.72)	0.82 (0.55, 1.23)	0.74 (0.53, 1.04)	0.38 (0.17, 0.84)	0.87 (0.58, 1.29)

Abbreviations: ACHIEVE = Aging and Cognitive Health Evaluation in Elders; OR = odds ratio; CI = confidence interval.
Estimated from weighted generalized estimating equations of falls occurring after randomization that were reported at each follow-up year (one, two, three). The model adjusted for the number of falls in the year before randomization, pair status, age, sex, race, recruitment source, field site, education, diabetes, hypertension, stroke, smoking status, CES-D score, SPPB balance score, , hearing severity, HHIE-S score, and the global cognition factor score. An interaction between time and each covariate was specified. The ACHIEVE study was conducted between 2017-2022 with recruitment from four US community sites.

eTable 3. Covariate-adjusted treatment effect of fall occurrence, fall recurrence, and injurious falls by treatment adherence, ACHIEVE study

Average daily hours of hearing aid use	Fall Occurrence OR (95% CI)	Fall recurrence OR (95% CI)	Injurious Falls OR (95% CI)
**Self-reported hours of hearing aid use**			
2 hours			
Year 1	0.58 (0.38, 0.89)	0.58 (0.38, 0.87)	0.58 (0.39, 0.88)
Year 2	0.97 (0.63, 1.47)	0.92 (0.61, 1.37)	0.89 (0.60, 1.33)
Year 3	0.80 (0.53, 1.20)	0.78 (0.52, 1.17)	0.83 (0.56, 1.24)
4 hours			
Year 1	0.62 (0.40, 0.98)	0.60 (0.38, 0.93)	0.61 (0.39, 0.94)
Year 2	1.05 (0.67, 1.66)	0.98 (0.63, 1.53)	0.98 (0.64, 1.49)
Year 3	0.77 (0.50, 1.18)	0.72 (0.47, 1.11)	0.78 (0.51, 1.20)
6 hours			
Year 1	0.68 (0.32, 1.44)	0.58 (0.29, 1.17)	0.58 (0.29, 1.15)
Year 2	1.27 (0.52, 3.12)	1.14 (0.59, 2.19)	1.10 (0.54, 2.24)
Year 3	0.81 (0.38, 1.75)	0.73 (0.38, 1.42)	0.80 (0.39, 1.66)
**Objectively measured hours of hearing aid use**			
2 hours			
Year 1	0.55 (0.38, 0.78)	0.56 (0.39, 0.79)	0.57 (0.40, 0.80)
Year 2	0.99 (0.69, 1.43)	0.96 (0.67, 1.36)	0.94 (0.67, 1.34)
Year 3	0.74 (0.51, 1.08)	0.71 (0.50, 1.03)	0.77 (0.54, 1.10)
4 hours			
Year 1	0.54 (0.36, 0.79)	0.54 (0.37, 0.80)	0.55 (0.37, 0.79)
Year 2	1.04 (0.71, 1.53)	1.01 (0.70, 1.47)	0.98 (0.68, 1.41)
Year 3	0.81 (0.55, 1.20)	0.76 (0.52, 1.12)	0.84 (0.58, 1.23)
6 hours			
Year 1	0.53 (0.33, 0.84)	0.53 (0.34, 0.84)	0.54 (0.34, 0.85)
Year 2	1.14 (0.73, 1.78)	1.08 (0.71, 1.65)	1.06 (0.69, 1.62)
Year 3	0.97 (0.61, 1.52)	0.90 (0.58, 1.41)	0.98 (0.63, 1.52)

Abbreviations: ACHIEVE = Aging and Cognitive Health Evaluation in Elders; OR = odds ratio; CI = confidence interval.
Treatment adherence defined as average daily duration of hearing aid use by self-report and device data logging. Estimated from weighted generalized estimating equations of falls occurring after randomization that were reported at each follow-up year (one, two, three). The model adjusted for the number of falls in the year before randomization, pair status, age, sex, race, recruitment source, field site, education, diabetes, hypertension, stroke, smoking status, CES-D score, SPPB balance score, hearing severity, HHIE-S score, and the global cognition factor score. An interaction between time and each covariate was specified. The ACHIEVE study was conducted between 2017-2022 with recruitment from four US community sites.

eTable4. Covariate adjusted intention to treat analysis of the 3-year rate of falls stratified by self-reported sex, ACHIEVE study

	Total<br>Mean (95% CI)	Female<br>Mean (95% CI)	Male<br>Mean (95% CI)
Control	1.98 (1.82, 2.15)	1.45 (1.23, 1.67)	2.33 (1.99, 2.66)
Intervention	1.45 (1.28, 1.61)	0.99 (0.79, 1.19)	1.03 (0.69, 1.38)
Difference	-0.54 (-0.77, -0.31)	-0.46 (-0.74, -0.18)	-1.29 (-1.76, -0.82)

Abbreviations: ACHIEVE = Aging and Cognitive Health Evaluation in Elders; CI = confidence interval. Estimated from weighted linear regression models that examined the average number of falls over three-years per participant. The covariate-adjusted model included the number of years of follow-up, the number of falls in the year before randomization, pair status, age, sex, race, recruitment source, field site, education, diabetes, hypertension, stroke, smoking status, CES-D score, SPPB balance score, hearing severity, HHIE-S score, and the global cognition factor score. The ACHIEVE study was conducted between 2017-2022 with recruitment from four US community sites.
